# Transcriptome‐wide analysis reveals GYG2 as a mitochondria‐related aging biomarker in human subcutaneous adipose tissue

**DOI:** 10.1111/acel.14049

**Published:** 2023-12-08

**Authors:** Mira Ham, Yeonju Cho, Tae‐Wook Kang, Taeyun Oh, Hyoung‐June Kim, Kyu‐Han Kim

**Affiliations:** ^1^ R&I Unit, Amorepacific Corporation Gyeonggi‐do Korea; ^2^ Department of Bioinformatics The Moagen Inc. Daejeon Korea; ^3^ Department of Internal Medicine, Institute of Gastroenterology Yonsei University College of Medicine Seoul Korea

**Keywords:** adipocyte, aging, computational biology, GYG2, mitochondria, subcutaneous adipose tissue, transcriptome, WGCNA

## Abstract

Subcutaneous adipose tissue (SAT), a vital energy reservoir and endocrine organ for maintaining systemic glucose, lipid, and energy homeostasis, undergoes significant changes with age. However, among the existing aging‐related markers, only few genes are associated with SAT aging. In this study, weighted gene co‐expression network analysis was used on a transcriptome of SAT obtained from the Genotype‐Tissue Expression portal to identify biologically relevant, SAT‐specific, and age‐related marker genes. We found modules that exhibited significant changes with age and identified *GYG2* as a novel key aging associated gene. The link between *GYG2* and mitochondrial function as well as brown/beige adipocytes was supported using additional bioinformatics and experimental analyses. Additionally, we identified PPARG as the transcription factor of GYG2 expression. The newly discovered *GYG2* marker can be used to not only determine the age of SAT but also uncover new mechanisms underlying SAT aging.

## Graphical abstract

An analysis of human subcutaneous adipose tissue, using WGCNA, revealed that glycogenin‐2 (GYG2) decreases with age, especially in females. The expression of GYG2 was found to be closely associated with biological age and adipocyte browning.

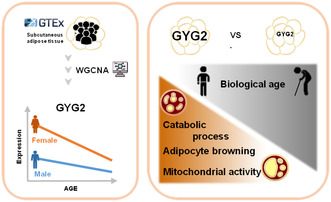

**Abbreviations**
ADSC: adipose‐derived stem cell
BMI: body mass index
DEG: differentially expressed gene
ECM: extracellular matrix
GO: gene ontology
GSEA: gene set enrichment analysis
GTEx: genotype‐tissue expression
GYG2: glycogenin‐2
MM: module membership
NES: normalized enrichment score
OCR: oxygen consumption rate
SASP: senescence‐associated secretory phenotype
SAT: subcutaneous adipose tissue
TPM: transcript per million
UMAP: uniform manifold approximation and projection
VAT: visceral adipose tissue
WGCNA: weighted gene co‐expression network analysis

## 1 INTRODUCTION

Subcutaneous adipose tissue (SAT) regulates whole‐body energy homeostasis and functions as a caloric reservoir and endocrine organ. As a caloric reservoir, SAT stores extra calories in the form of lipids and utilizes them when needed. As an endocrine organ, adipose tissue releases adipokines, such as adiponectin and leptin, which regulate various processes, including appetite, glucose homeostasis, insulin sensitivity, inflammation, and tissue repair (Hassan et al., 2014). SAT undergoes significant physiological and morphological changes as it ages (Varghese et al., 2021), including lipid redistribution from the subcutaneous to the visceral adipose tissue (VAT), decline in beiging upon cold exposure (Berry et al., 2017), dysregulation of adipokines, and increased low‐grade chronic inflammation. Alterations in the amount, composition, and physiological function of adipose tissue induce insulin resistance and ectopic lipid accumulation (Ahmed et al., 2021), which contribute to the development of metabolic disorders, such as Type 2 diabetes, cardiovascular disease, and hepatic steatosis.

Senescence of cells and decline in the expression of their marker genes, such as cell cycle‐related and senescence‐associated secretory phenotype (SASP) genes, are increased in various tissues of older individuals (Saul et al., 2022). Similar to that in other tissues, senescent cells accumulate in the adipose tissue of older individuals (Rouault et al., 2021), but changes in the expression of senescence markers are not evident. *CDKN2A* (p16^Ink4a^), a cell cycle‐related gene, is not sufficiently expressed in human adipose tissue. Another cell cycle‐related gene, *CDKN1A* (p21^CIP1^), is weakly correlated with age (Hudgins et al., 2018). SASPs are also markers of senescence and show a clear correlation with age; however, they cannot be considered specific markers of aging owing to their strong association with body mass index (BMI) (Varghese et al., 2021).

Weighted gene co‐expression network analysis (WGCNA) is a bioinformatics application that uses the coordinated co‐expression of genes to help gain a comprehensive understanding of the transcriptome and uncover the underlying cellular processes (Zhang & Horvath, 2005). WGCNA enables evaluation of module associations with phenotypic traits using network properties as well as identification of hub genes. We used WGCNA to analyze the SAT transcriptome from Genotype‐Tissue Expression (GTEx) portal, which provides a large amount of data across various ages, to identify SAT‐specific and age‐related marker genes. The aging marker discovered using these methods will not only measure the aging of SAT more accurately, but will also serve as a cornerstone for understanding the aging of SAT.

## 2 MATERIALS AND METHODS

### 2.1 WGCNA

Fully processed, filtered, and normalized gene expression data of SAT (442 samples) were downloaded from the GTEx Project portal version 7.

The R package WGCNA (https://horvath.genetics.ucla.edu/html/Co‐expressionNetwork/Rpackages/WGCNA/index.html) (Langfelder & Horvath, 2008) was used to analyze the co‐expression of genes in SAT. Among the 442 subjects from the GTEx database, 33 outliers were excluded (Figure S1a). After the genes with low expression were removed, 16,281 were used for analysis. A soft threshold (power) of 9, a signed topological overlap matrix, and a minimum module size of 30 were selected to construct a scale‐free network and module detection (Figure S1b,c). A module–trait correlation analysis was performed (Figure S1d).

### 2.2 Analysis of expression data

Samples were grouped based on high (GYG2_H) and low (GYG2_L) *GYG2* expression levels. The R package DESeq2 was used to compare differences between the GYG2_L and GYG2_H groups (*n* = 20). Genes with a *p* value ≤0.05 and log2 fold change |log2FC| ≥ 2 were identified as differentially expressed genes (DEGs). For Gene Ontology (GO) enrichment analysis and gene set enrichment analysis (GSEA), the gene list of specific modules from WGCNA and the gene list from the DEG analysis were analyzed using the R package clusterProfiler (Yu et al., 2012).

For RNA age calculations, individuals with low or high GYG2 levels were selected from each decade group (*n* = 10 for individuals in their 20s and 30s, *n* = 15 for those in their 40s, *n* = 30 for those in their 50s, and *n* = 20 for those in their 60s). RNA ages were calculated using the R package RNAAgeCalc (Ren & Kuan, 2020), which was developed to calculate transcriptional age from RNA‐sequencing (RNA‐seq) data. We calculated the RNA ages using raw count data and the GenAges age signatures.

Samples from individuals in their 50s and 60s were sorted according to the mRNA expression levels of *CIDEA* and *GYG2*. For the browning probability potential, the raw read counts of CIDEA_50s_H, CIDEA_50s_L, CIDEA_60s_H, CIDEA_60s_L, GYG2_50s_H, GYG2_50s_L, GYG2_60s_H, and GYG2_60s_L were applied in the ProFAT online tool (Cheng et al., 2018).

The mitochondrial DNA copy number was estimated according to a previous study (Cho, Yoo, & Seo, 2018). Sixteen mitochondrial genes (*MT‐RNR1*, *MT‐RNR2*, *MT‐ND1*, *MT‐ND2*, *MT‐CO1*, *MT‐TS1*, *MT‐CO2*, *MT‐ATP8*, *MT‐ATP6*, *MT‐CO3*, *MT‐ND3*, *MT‐ND4*, *MT‐ND4L*, *MT‐ND5*, *MT‐ND6*, and *MT‐CYB*) with average mapped reads exceeding 100 were subjected to the analysis. We calculated the mitochondrial DNA copy number using the mean expression values of the 16 mitochondrial RNAs. Associations between aging and mitochondrial RNA expression were measured using one‐way analysis of variance (ANOVA) for global analysis and Scheffe's test for post hoc analysis.

The median gene‐level transcript per million (TPM) by tissue of the RNA‐Seq data downloaded from the GTEx portal was used to calculate gene expression. To assess relative expression levels, the TPM value of each tissue was divided by the average TPM value of the entire tissue.

A previously published snRNA‐Seq dataset (ArrayExpress: E‐MAB‐8564) was analyzed using Seurat package (Satija et al., 2015) in R. Cells that had more than 0.5% expression on mitochondrial genes and fewer than 150 features were removed. DoubletFinder (McGinnis et al., 2019) was used to exclude potential doublets. The *Normalization* and *FindVariableFeatures* functions were used for preprocessing. Clusters were determined using *FindNeighbors* and *FindClusters* in Seurat after generating Uniform Manifold Approximation and Projection (UMAP) data using *RunUMAP*. FeaturePlot of *GYG2* and brown/beige marker genes were depicted using *FeaturePlot*.

EnrichR, the extensive gene set enrichment analysis tool (Kuleshov et al., 2016), was used to find transcription regulators for GYG2. We used 73 hub genes from the black module (with gene significance >0.2 and module membership (MM) >0.8) as our query. Five ChIP‐Seq databases (ChEA 2022, ENCODE and ChEA Consensus TFs, ARCHS4 TFs Coexp, TF Perturbations Followed by Ex, and TRRUST Transcription Factors 2019) were used for analysis. Eleven transcription factors that were statistically significant (adj. *p*‐value <0.05) and found in more than two databases were identified.

A previously published RNA‐Seq dataset (GSE13457) was used to examine the expression of *GYG2* mRNA in forskolin‐treated adipocytes. The TPM value of *GYG2* mRNA was used to construct a box plot.

### 2.3 Cell culture

Adipose‐derived stem cells (ADSCs; Lonza, Basel, Switzerland) were cultured in ADSC growth medium (ADSCGM; Lonza) containing supplements with growth factors (ADSCGM‐SingleQuotsTM Kit; Lonza) at 37°C in a 5% CO_2_ incubator.

In the replicative senescent ADSC model, an equal number (10^5^ cells) of both early passage (young: passages 3–5) and late passage (old: passages 14–16) ADSCs were used for RNA isolation.

For adipocyte differentiation, ADSCs were cultured until they reached 100% confluence. After 24 h, ADSCs were incubated with 10% fetal bovine serum (FBS)‐supplemented Dulbecco's modified Eagle's medium (DMEM) containing 1 μg/mL of insulin (Cat. 11 376 497 001, Roche, Basel, Switzerland), 520 μM 1‐methyl‐3‐isobutyl‐xanthine (Cat. I5879, Sigma‐Aldrich), 1 μM dexamethasone (Cat. D1756, Sigma‐Aldrich), and 1 M troglitazone (Cat. T2573, Sigma‐Aldrich) for 1 week. In the second week, the cells were cultured using the same medium used in the first week, but without 1‐methyl‐3‐isobutyl‐xanthine and dexamethasone.

The aging adipocyte model was prepared according to a previous report (Zoico et al., 2019) with modifications. After 2 weeks of differentiation, adipocytes were maintained with 10% FBS‐supplemented DMEM for 2 or 4 weeks.

Beige adipocytes were differentiated from ADSCs, according to a previous report (Singh et al., 2020).

For small interfering (si)RNA transfection, ADSCs were electroporated with either siNC or siGYG2 (Silencer Select predesigned siRNA Cat. #4392420; Thermo Fisher Scientific) and plated at a density greater than 70% confluence to allow for further expansion. Adipogenesis was induced when the cells achieved confluence.

To investigate the effects of PPARG ligands, fully differentiated adipocytes were exposed to either rosiglitazone (10 μM) or troglitazone (2 μM) for 3 days.

### 2.4 Determination of lipid accumulation

To examine lipid accumulation, the cells were fixed with paraformaldehyde and stained with Oil Red O. Four images of each group were used for the analysis of the relative mean area of lipid droplets using ImageJ (https://imagej.nih.gov/ij/download.html). The lipid droplets were outlined and their area was measured.

### 2.5 Reverse transcription‐quantitative polymerase chain reaction (RT‐qPCR)

Total RNA was isolated using the TRIzol reagent (Cat. 15,596,018, Thermo Fisher Scientific), and then 1 μg of RNA was reverse‐transcribed to generate cDNAs using the RevertAid First Strand cDNA Synthesis Kit (Cat. K1622, Thermo Fisher Scientific) according to the manufacturer's instructions. Quantitative real‐time PCR was performed using TaqMan probes (Thermo Fisher Scientific), TaqMan Universal Master Mix (Cat. 4440038, Thermo Fisher Scientific), and a 7500 Fast Real‐Time PCR System (Thermo Fisher Scientific). Reactions were performed using the following cycling conditions: 95°C for 10 min, then 40 cycles consisting of 95°C for 15 s and 60°C for 1 min. Cycle threshold values for mRNA were normalized against levels of the endogenous gene *GAPDH* or *RPL13A*. The TaqMan probes are listed in Table S1.

### 2.6 Mitochondrial bioenergetics analysis

Oxygen consumption rate (OCR) from adipocytes was measured using a Seahorse XFe96 Analyzer (Agilent). ADSCs electroporated with either siNC or siGYG2 were seeded and differentiated to adipocytes in XFe96 Microplates (Cat. 103794‐100, Agilent). The culture medium was replaced with XF assay medium supplemented with 25 mM glucose (Cat. 103577‐100, Agilent), 4 mM glutamine (103579‐100, Agilent), and 1 mM pyruvate (Cat. 103578‐100, Agilent), and cells were incubated in the non‐CO_2_ incubator for 45 min before assessment. Mitochondrial function was evaluated using Seahorse XF Cell Mito Stress Test kit (Cat. 103015‐100, Agilent) at baseline and after sequential addition of oligomycin, Carbonyl cyanide‐4 (trifluoromethoxy) phenylhydrazone (FCCP), and a mixture of rotenone and antimycin A at final concentrations of 1.5, 0.5, and 0.5 μM respectively. Data are expressed as the rate of oxygen consumption in pmol/min normalized to cell protein in individual wells, as determined by a Pierce BCA protein assay (Cat. 23228, Thermo Fisher Scientific).

### 2.7 Statistical analysis

Data are presented as representative examples or as mean ± SD of two or more experiments. *p* values were obtained using appropriate statistical methods including ANOVA, Student's *t* test, and Wilcoxon test according to the experiments.

## 3 RESULTS

### 3.1 Age‐related modules identified using WGCNA

**FIGURE 1:**
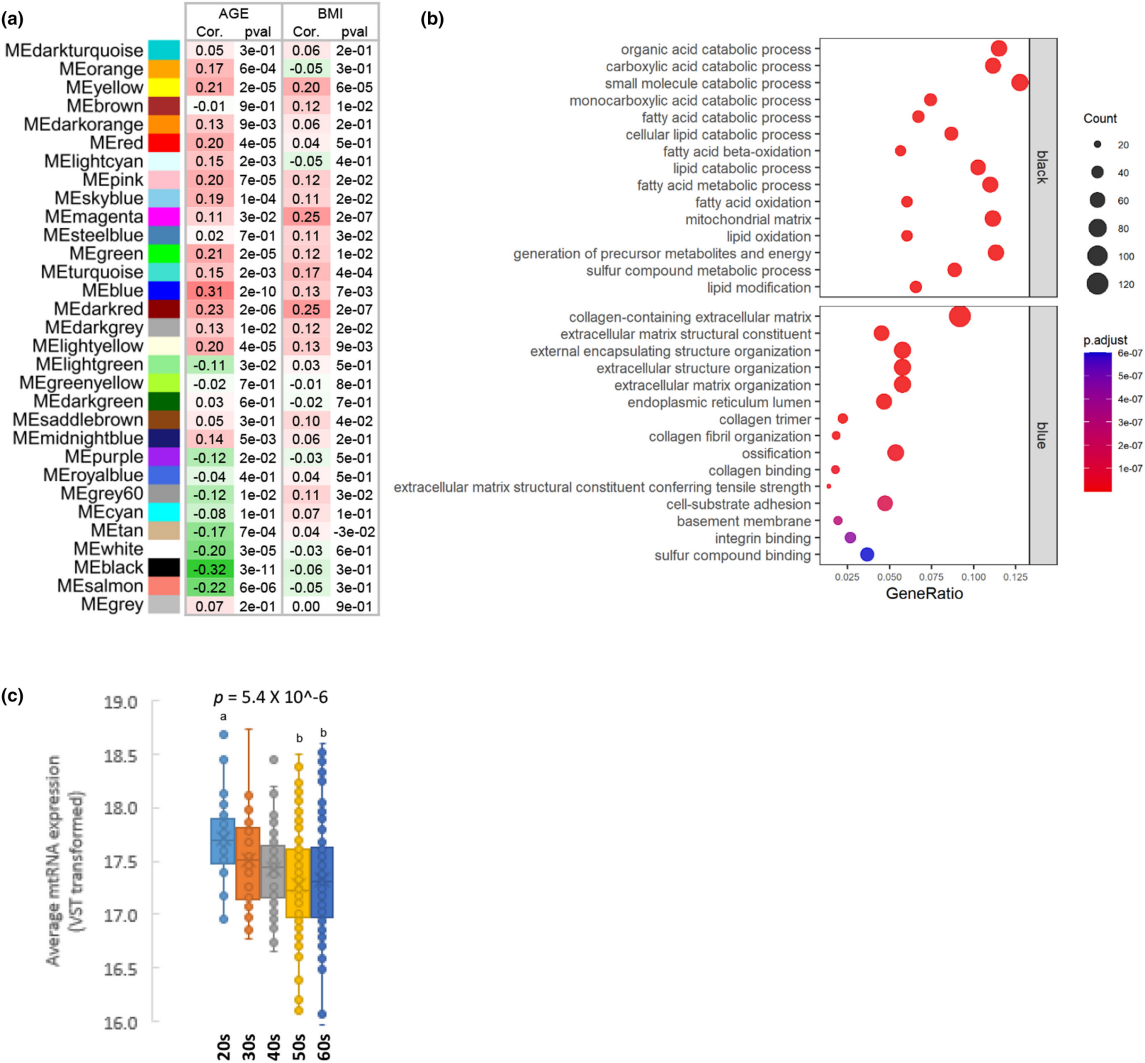
SAT age‐related modules identified using WGCNA. (a) Module–trait relationship of constructed modules. This heatmap shows the correlation of module eigengenes with age and BMI. Corresponding correlation coefficients and *p* values are provided. Significant values are presented in bold font. (b) GO enrichment analysis of the black module, which is most negatively correlated with age, and the blue module, which is most positively correlated with age. The top 15 GO terms are shown. Gene ratio = number of genes that were enriched on the given pathway/total number of genes on the given gene set. The adjusted *p* value illustrates the significance. (c) Box plots of average mitochondrial RNA expression of decade groups compared using ANOVA for global analysis and Scheffe's test for post hoc analysis. GO, Gene Ontology; SAT, subcutaneous adipose tissue; WGCNA; weighted gene‐coexpression network analysis.

To identify co‐expression networks that correlated with age, we analyzed SAT RNA‐seq data (*n* = 409) from GTEx using WGCNA (Figure S1). Among 31 modules, certain modules, such as the dark red module, exhibited a high correlation with both age and BMI, whereas other modules, specifically the blue and black modules, exhibited a higher correlation with age than with BMI (Figure 1a). The dark red module was particularly enriched for genes related to leukocyte cell–cell adhesion, T cells, and lymphocytes. The blue module was enriched for genes related to the extracellular matrix (ECM), such as extracellular structure organization, collagen fibril organization, and cell–substrate adhesion. The black module was enriched for genes related to adipose tissue metabolism, such as the organic acid catabolic process and fatty acid catabolic process (Figure 1b and Table S2), which is consistent with the results of previous studies showing metabolic decline with age in adipose tissue (Caso et al., 2013; Stout et al., 2014). Because the top 15 terms analyzed from the black module were closely associated with mitochondrial function, we estimated mitochondrial DNA copy number, which is directly correlated with mitochondrial function (Castellani et al., 2020; Cho, Yoo, & Seo, 2018; Cho, Yoo, Song, et al., 2018). The mitochondrial DNA copy number estimated using mitochondrial RNA expression were negatively correlated with age (Figure 1c).

### 3.2 Key genes in the black module

**FIGURE 2:**
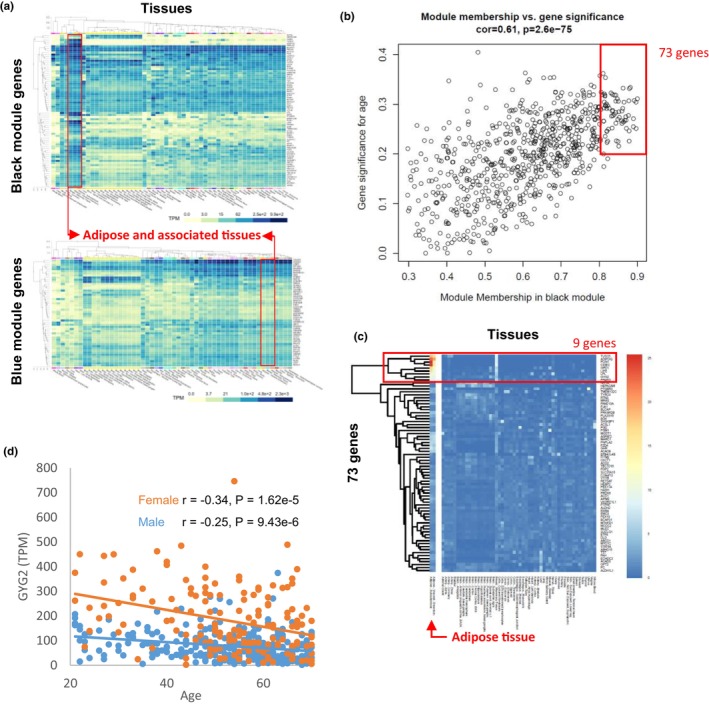
Identification of aging‐associated key genes in SAT. (a) Expression distribution of key genes in the black and blue modules across various tissues. Genes with high MM (>0.8) and high gene significance (>0.2) were selected for analysis. Expression of genes in SAT, VAT, and mammary tissue is marked with red boxes. The heatmap is derived from the GTEx portal. (b) The scatterplot was generated to depict correlations between MM and gene significance values for the black module. Genes with high MM (>0.8) and high gene significance (>0.2) were selected and marked with a red box. (c) The heatmap shows the expressions of 73 genes in various tissue. Nine genes that are highly (TPM >50) and specifically expressed in adipose tissues are marked with a red box. (d) The scatterplot of *GYG2* mRNA expression according to age in adipose tissue. The solid orange and blue colors indicate female and male, respectively. r represents the Pearson correlation coefficient between gene expression and age. GTEx, Genotype‐Tissue Expression; MM, module membership; SAT, subcutaneous adipose tissue; TPM; transcript per million; VAT, visceral adipose tissue.

Within each module, we examined the tissue distribution of hub genes (gene significance >0.2 and module membership (MM) >0.8). Interestingly, all the hub genes in the black module were abundantly expressed in adipose tissue, and approximately half of them were specifically expressed only in the SAT and related tissues, such as VAT and mammary tissues (Figure 2a). However, the hub genes in the blue module showed low expression or no specificity between tissues. These results suggest that the black module may represent an aging phenotype specific to SAT.

To identify novel biomarkers of the SAT‐specific aging phenotype, we further analyzed the genes in the black module. Genes with significance (>0.2), MM (>0.8) (Figure 2b), and adipose tissue‐specificity (Figure 2c) were considered. Thus, nine genes (*ADIPOQ*, *CIDEC*, *GPD1*, *GYG2*, *LIPE*, *PLIN1*, *TRARG1*, *LPL*, and *PPARG*) were selected and their expression at the protein level was verified using immunohistochemistry data, excluding LPL (The HPA Tissue Atlas, 2023) (Figure S2). Previous studies have investigated the functions of *ADIPOQ*, *CIDEC*, *GPD1*, *LIPE*, *PLIN1*, *TRARG1*, *LPL*, and *PPARG* in adipose tissue, and some of these genes have also been associated with aging. *GYG2*, which has not been extensively studied in relation to adipose tissue or aging, encodes glycogenin‐2, a self‐glucosylating protein that plays a key role in the initiation of glycogen biosynthesis (Mu & Roach, 1998). We observed distinct sex‐specific expression patterns for *GYG2* mRNA, with significantly higher levels in females compared with that in males (Figure 2d). However, no such reduction was observed in VAT (Figure S2a). Additionally, no significant correlation between *GYG2* mRNA expression and BMI was observed in either type of adipose tissue (Figure S2b,c), suggesting that *GYG2* is a BMI‐independent marker. Given its SAT‐specific, and BMI‐independent expression, *GYG2* is a strong candidate marker gene for SAT aging.

### 3.3 Negative correlation between *GYG2* expression and aging

**FIGURE 3:**
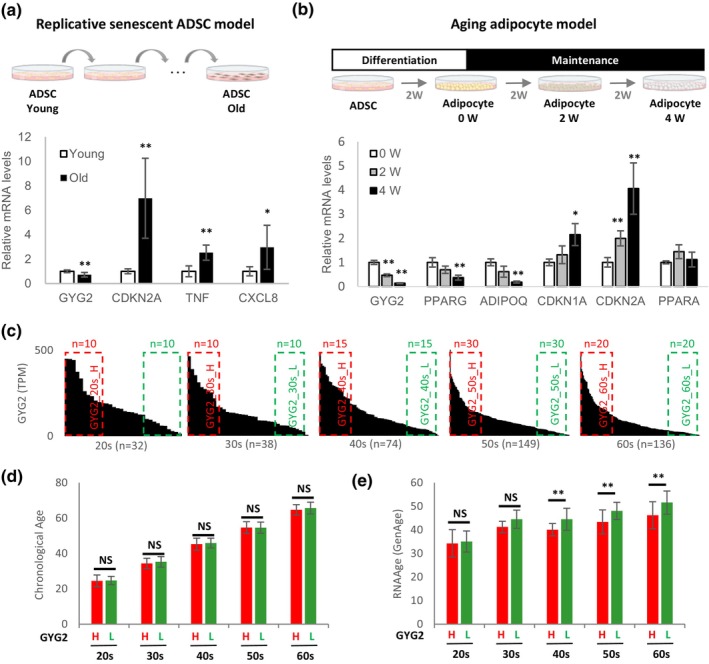
Negative relationship between *GYG2* expression and age. (a) mRNA levels of *GYG2*, *CDKN2A*, *TNF*, and *CXCL8* in replicative senescent ADSCs. Senescence of ADSCs was induced by prolonged subculture. The values are reported as means ± SD (*n* = 6). (b) mRNA levels of *GYG2*, *PPARG*, *ADIPOQ*, *CDKN1A*, *CDKN2A*, and *PPARA* using an in vitro model of aging adipocytes. After differentiation, the cells were maintained for an additional 2 or 4 weeks to induce senescence. The values are reported as means ± SD (*n* = 3). (c) TPM of *GYG2* in descending order in each decade of adipose tissue by age. Samples with high and low GYG2 expression are marked with red and green boxes, respectively (*n* = 10 for individuals in their 20s and 30s, *n* = 15 for those in their 40s, *n* = 30 for those in their 50s, *n* = 20 for those in their 60s). (d) Chronological ages of GYG2_H and GYG2_L groups in each decade group. The values are reported as means ± SD. NS: not significant. (e) RNA age of GYG2_H and GYG2_L samples. The values are reported as means ± SD. NS: not significant. *p < 0.05 and **p < 0.01 using the Student's *t* test. ADSCs, adipose‐derived stem cells; TPM; transcript per million.

Subsequently, the expression of *GYG2* in two in vitro aging models was investigated utilizing human ADSCs. The first model involved replicative senescence induced by prolonged passaging of undifferentiated ADSCs (Xu et al., 2015). The second model entailed maintaining adipocytes for several weeks after inducing adipocyte differentiation from ADSCs, leading to the induction of cellular aging (Zoico et al., 2019). In the replicative senescent ADSC model, *GYG2* expression significantly decreased while *CDKN2A*, *TNF*, and *CXCL8* expression increased (Figure 3a). In the aging adipocyte model, *GYG2* expression significantly reduced, while *CDKN1A* and *CDKN2A* expression increased (Figure 3b). No changes in the lipid contents were observed (Figure S4a,b).

To further investigate the relationship between *GYG2* mRNA expression and aging, we measured biological age using the recently developed RNA‐sequencing based transcriptomic clock tool “RNAAgeCalc,” which calculates RNA age. We examined whether there is a difference in biological age depending on the *GYG2* expression in subjects of the same chronological age. First, all samples were classified based on the decade. Subsequently, within each decade, samples were sub‐grouped based on high (GYG2_H) and low GYG2 (GYG2_L) mRNA expression (Figure 3c). There was no significant difference in the chronological age depending on *GYG2* expression in all decades (Figure 3d). However, the RNA ages were significantly decreased in GYG2_H compared to those in GYG2_L in the 40s or older group (Figure 3e). The data indicate that *GYG2*, a marker derived based on chronological age, is also associated with biological age.

### 3.4 Characteristics of GYG2 based on DEG analysis

**FIGURE 4:**
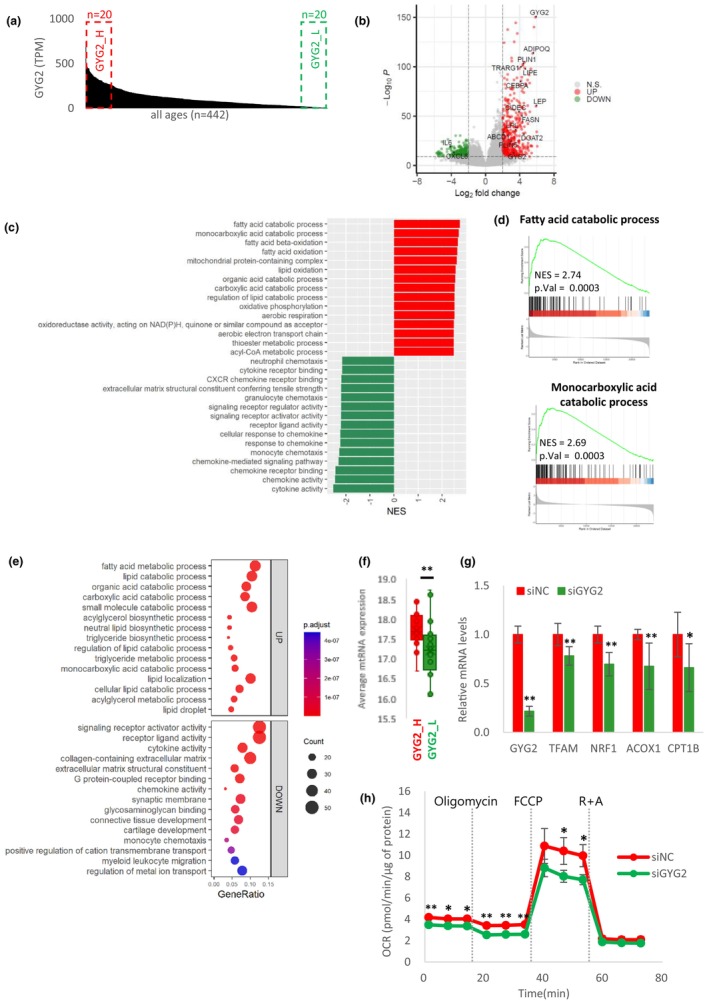
Characterization of *GYG2*. (a) TPM of *GYG2* in descending order in adipose tissue. Samples with high and low GYG2 expression are marked with red and green boxes, respectively. (b) Volcano plot of differentially expressed genes (DEGs) between GYG2_H and GYG2_L. Gray, green, and red represent non‐significant, downregulated, and upregulated genes, respectively. (c) GSEA using DEGs between GYG2_H and GYG2_L. The top 15 enriched GO terms are presented in the bar plot. Red and green indicate the pathways enriched in GYG2_H and GYG2_L, respectively. (d) GSEA plots of fatty acid and monocarboxylic acid catabolic processes. The normalized enrichment score (NES) and the *p* value are indicated in the insert. (e) GO enrichment analysis using DEGs between GYG2_H and GYG2_L. The top 15 GO terms are shown in the dot plot. Gene ratio = number of genes that were enriched on the given pathway/total number of genes on the given gene set. The adjusted *p* value illustrates the significance. (f) Average mitochondrial RNA expression of GYG2_L and GYG2_H groups were compared. **p < 0.01 using a Wilcoxon test. (g) The mRNA levels of *GYG2*, *TFAM*, *NRF1*, *ACOX1*, *and CPT1* in siNC or siGYG2 transfected adipocytes. The values are reported as means ± SD (*n* = 6). (h) The oxygen consumption rate (OCR) of adipocytes transfected with siNC or siGYG2. Mitochondrial metabolic potential was measured using a Seahorse Bioanalyzer and determined by the sequential additions of oligomycin, FCCP, and a mixture of rotenone and antimycin A (R + A). Data are representative of two independent experiments. The values are reported as means ± SD (*n* = 3 for siNC and *n* = 4 for siGYG2). *p < 0.05 and **p < 0.01 using the Student's *t* test. DEGs, differentially expressed genes; FCCP, Carbonyl cyanide‐4 (trifluoromethoxy) phenylhydrazone; GO, Gene Ontology; GSEA, gene set enrichment analysis; TPM, transcript per million.

To further characterize the function of GYG2 in adipose tissue, we compared the GYG2_H and GYG2_L gene expression profiles (Figure 4a). A volcano plot was constructed based on the list of DEGs (Table S3) (Figure 4b). The expression of genes related to lipid metabolism, such as *ADIPOQ*, *LIPE*, and *DGAT2*, was significantly upregulated in GYG2_H compared with that in GYG2_L. In contrast, the expression of *IL6* and *CXCL8*, which are involved in inflammatory responses, was downregulated in GYG2_H compared with that in GYG2_L. Correlation analysis between *GYG2* and adipokines confirmed the significant association of *GYG2* with *ADIPOQ*, *IL6*, and *CXCL8* (Figure S2d,e).

We also performed GSEA (Figure 4c,d) and GO enrichment analysis (Figure 4e). In both analyses, fatty acid and monocarboxylic acid catabolic processes were enriched in the GYG2_H group. Notably, GSEA showed enrichment of terms related to mitochondria, including mitochondrial protein‐containing complex, oxidative phosphorylation, aerobic respiration, and aerobic electron transport chain. This result implies an increase in mitochondrial content and/or activity with increased *GYG2* expression. Consistent with this analysis, the GYG2_H group showed higher mitochondrial RNA expression levels than those shown by the GYG_L group (Figure 4f).

Next, we performed knockdown experiments using a siRNA against *GYG2* to evaluate the effect of GYG2 on mitochondria. The transfection of siGYG2 successfully decreased *GYG2* mRNA expression in differentiated adipocyte. Knockdown of *GYG2* led to decreased expression of *TFAM*, a crucial regulator of mitochondrial DNA replication and transcription. Additionally, the mRNA expression of *NRF1*, *ACOX1*, and *CPT1*, all of which are localized within the mitochondria and involved in mitochondrial fatty acid oxidation, was found to be downregulated upon *GYG2* knockdown (Figure 4g). To further analyze the functional difference in mitochondria, we assessed the OCR which mainly reflects the levels of mitochondrial oxidative phosphorylation activity. Consistent with decreased expression of mitochondrial genes, *GYG2* knockdown led to an overall decrease in OCR. Moreover, a major decrease in OCR was observed following the addition of the uncoupling agent FCCP, indicating a defect in maximal respiratory capacity upon *GYG2* knockdown (Figure 4h). Taken together, these results suggest that *GYG2* expression levels contribute to mitochondrial catabolic activity, which plays a major role in SAT aging.

### 3.5 Association between GYG2 and brown/beige adipocytes

Brown/beige adipocytes are specialized adipocytes responsible for calorie burning through non‐shivering thermogenesis, a process that involves fatty acid and glucose catabolism. (Singh et al., 2021; Whitehead et al., 2021). Previous studies have reported the loss of these cells with age (Berry et al., 2017; Rogers et al., 2012). Therefore, the high ranking of fatty acid and glucose catabolism in GSEA and GO enrichment analysis is particularly notable. Additionally, the pivotal role of glycogen metabolism, which involves GYG2, in the lipid droplet formation of brown adipocytes (Mayeuf‐Louchart et al., 2019), implies a significant role for GYG2 in the function of brown/beige adipocytes.

**FIGURE 5:**
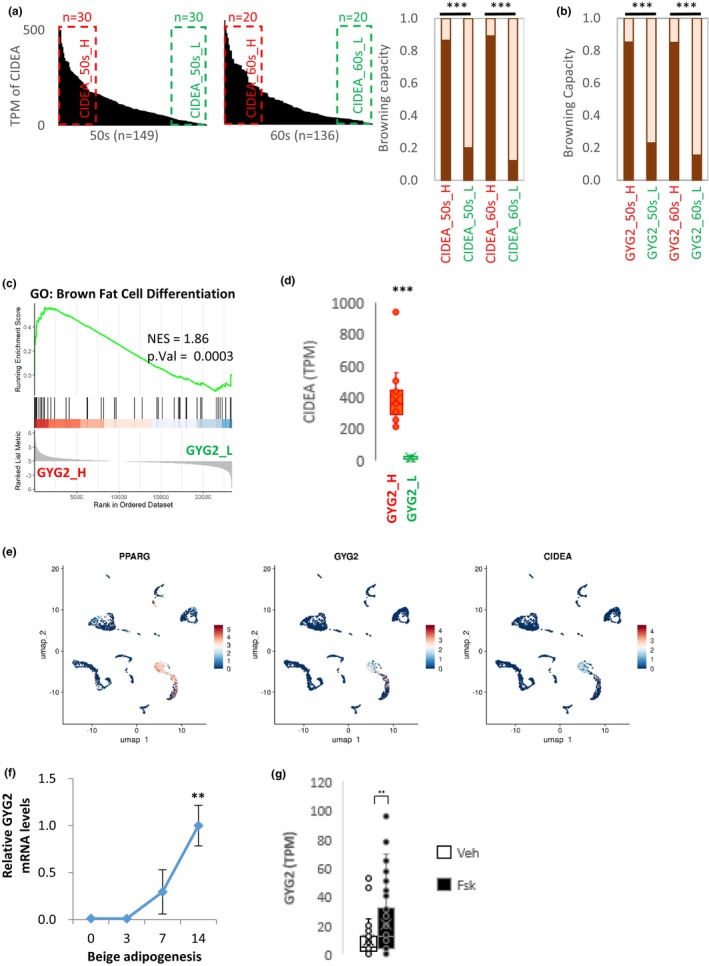
Function of GYG2 in adipocyte browning. (a) TPM of *CIDEA* in descending order in adipose tissue samples from individuals in their 50s and 60s. Samples with high and low *CIDEA* expression are marked with red and green boxes, respectively (*n* = 30 for individuals in their 50s, *n* = 20 for those in their 60s). The browning capacities of each group (CIDEA_50s_H, CIDEA_50s_L, CIDEA_60s_H, and CIDEA_60s_L) were calculated using ProFat. The values are reported as means. (b) The browning capacity of each group as defined in Figure 3c (GYG2_50s_H, GYG2_50s_L, GYG2_60s_H, and GYG2_60s_L) was calculated using ProFat. (c) GSEA plot of enrichment in “brown fat cell differentiation” signature in GYG2_H and GYG2_L. The NES and the *p* value are indicated in the insert. (d) *CIDEA* mRNA of GYG2_H and GYG2_L groups were compared. (e) Expression patterns of *PPARG*, *GYG2*, and *CIDEA* at the single‐nucleus level in human BAT using a previously published dataset (ArrayExpress: E‐MAB‐8564). (f) mRNA expression of *GYG2* during beige adipogenesis. The expression levels during 14 days of adipogenic differentiation were determined. The values are reported as means ± SD (*n* = 3). (g) Box plot showing the expression level of *GYG2* in forskolin‐stimulated adipocytes in which RNA‐sequencing data from GSE134570 were used. The values are reported as means. ***p < 0.001 and **p < 0.01 using the Student's *t* test. GSEA, gene set enrichment analysis; NES, normalized enrichment score; TPM, transcript per million.

To investigate the association of GYG2 with adipocyte browning, we used ProFat (Cheng et al., 2018), a neural network‐based tool, to compute the thermogenic potential (brown adipocyte content) using transcriptome data. As a positive control, the influence of *CIDEA* (a marker gene for brown/beige adipose tissue) on thermogenic potential was analyzed. Samples from individuals in their fifties and sixties were sorted according to the mRNA levels of *CIDEA*, and samples with high and low *CIDEA* expression were then selected as CIDEA_50s_H, CIDEA_50s_L, CIDEA_60s_H, and CIDEA_60s_L, respectively (Figure 5a). The thermogenic potentials of CIDEA_50s_H and CIDEA_60s_H were significantly higher than those of CIDEA_50s_L and CIDEA_60s_L, respectively. The thermogenic potential of GYG2 was calculated in the same manner (Figure 5b). The thermogenic potentials of GYG2_50s_H and GYG2_60s_H were significantly higher than those of GYG2_50s_L and GYG2_60s_L, respectively, suggesting that adipose tissue with high GYG2 expression has high brown adipocyte activity. In line with these results, the term “brown fat cell differentiation” was enriched in GYG2_H, with a high normalized enrichment score (NES) (Figure 5c). In addition, the expression of *CIDEA* was significantly higher in the GYG2_H group than in the GYG2_L group (Figure 5d). Furthermore, reanalysis of a previously published single nucleus RNA‐seq study (Sun et al., 2020) has revealed that GYG2 serves as a marker for brown adipocytes, and the its expression patterns align well with that of CIDEA, a known marker gene for brown or beige adipocytes (Figure 5e).

To investigate the expression of GYG2 in beige adipocytes, ADSCs were induced into beige adipocytes using a previously described protocol (Singh et al., 2020), which mimics the development of new beige adipocytes from adipocyte progenitor cells. The expression of GYG2 increased rapidly during beige adipogenesis (Figure 5f). Additionally, the expression of *GYG2* was analyzed using RNA‐Seq data from forskolin‐treated adipocytes in GSE134570 (Min et al., 2019), which mimics the transdifferentiation of beige adipocytes from white adipocytes in vitro (Lemenager et al., 2020; Whitehead et al., 2021). *GYG2* expression was significantly higher in forskolin‐treated adipocytes than in vehicle‐treated cells (Figure 5g). Based on these results, we concluded that GYG2 expression is increased in beige adipocytes.

### 3.6 Identification and validation of PPARG as a key regulator of GYG2 expression

**FIGURE 6:**
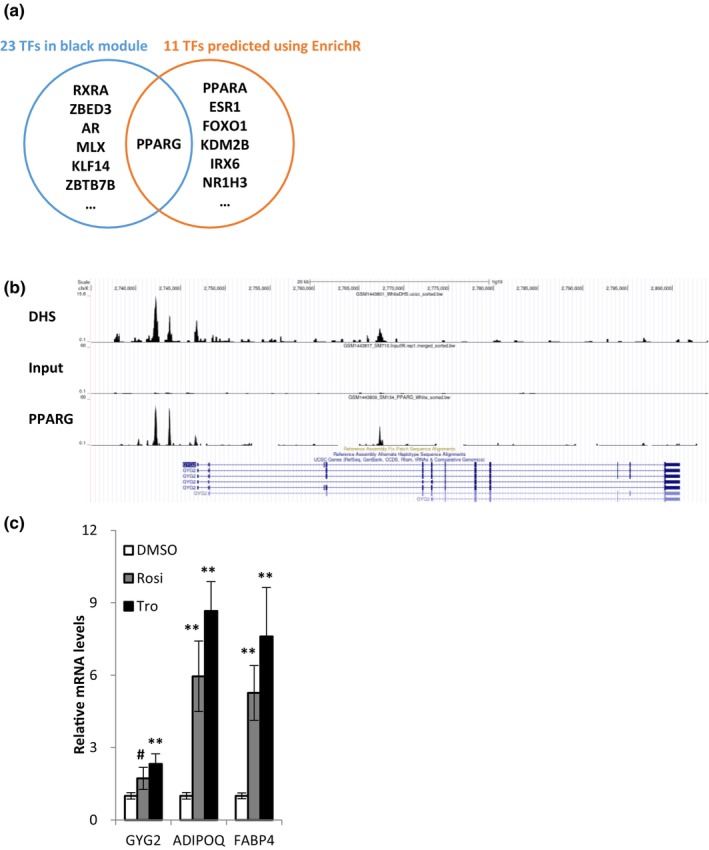
PPARG as a transcription factor regulating GYG2 expression. (a) Venn diagram of transcription factors potentially regulating GYG2. (b) Screenshot of the UCSC Genome Browser showing tracks for DNase I hypersensitive site (DHS), input, and PPARG around the *GYG2* gene. Compared to input track, PPARG track shows significantly enriched signals in DHSs. ChIP‐Seq datasets are available from GEO with the following accession numbers: GSE59703. (c) mRNA levels of *GYG2*, *ADIPOQ*, and *FABP4* in PPARG ligand treated adipocytes. The values are means ± SD (*n* = 3). #*p* = 0.059 and **p < 0.01 using the Student's *t* test. Rosi, rosiglitazone; Tro, troglitazone.

We sought to identify transcription factors capable of modulating the expression of GYG2 based on two specific criteria: transcription factors displaying considerable correlation with GYG2 in adipose tissue and those that are ranked high as GYG2 regulators within bioinformatics databases. First, the transcription factors that exhibited a significant correlation with *GYG2* expression were found in the black module of the WGCNA, which is where GYG2 resides. Using two transcription factor databases (Lambert et al., 2018; Lizio et al., 2019), we identified 23 transcription factors from a total of 727 genes within the black module (Table S4). Next, we employed EnrichR, an extensive gene set enrichment analysis tool (Kuleshov et al., 2016), which ranks transcription factors associated with query genes. Using 73 hub genes of the black module, a list of 11 transcription factors was obtained (Table S5). Comparing the two lists of transcription factors, we selected the common transcription factor, which was PPARG (Figure 6a). To validate our predictive analysis, we investigated results of the PPARG ChIP assay (Loft et al., 2015) and confirmed that PPARG directly binds to the promoter of *GYG2* (Figure 6b). Additionally, treatment of PPARG ligands (rosiglitazone and troglitazone) in adipocytes increased the expression of *GYG2* mRNA significantly (Figure 6c). Overall, the findings demonstrate that PPARG is a critical transcription factor modulating the expression of *GYG2*.

## 4 DISCUSSION

In the present study, the SAT transcriptome was analyzed using WGCNA to identify aging‐related marker genes. We identified an age‐related black module and its hub genes. Among the hub genes, *GYG2* was identified as a novel marker for SAT aging. Its functions were further evaluated using various bioinformatics analyses and in vitro experiments.

In recent decades, research has primarily focused on global aging‐related markers that encompass all body tissues. However, there is a growing need for research on tissue‐specific aging‐related markers (Xu et al., 2022). Because the aging rate and process vary across different tissues, accurately assessing tissue aging using global markers that apply universally can be challenging. (Nie et al., 2022). SAT did not express a significant number of global aging markers identified in previous studies, such as the molecular signatures of aging (de Magalhaes et al., 2009), SenMayo (Saul et al., 2022), and CellAge (Avelar et al., 2020) (Figure S3a–c). Tissue‐specific aging markers provide a comprehensive understanding of the underlying mechanisms specific to each tissue and have the potential to facilitate the development of more precise diagnostic tools for age‐related diseases that specifically affect certain tissues.

Previous studies that aimed to identify aging‐related marker genes in adipose tissue have provided limited information regarding aging signatures. Studies conducted in model organisms, such as rodents and monkeys, have demonstrated that PPARG, SREBP1, C/EBPs, LPL, and GLUT4 levels decrease with age (Hotta et al., 1999; Karagiannides et al., 2001; Xu et al., 2018). Recently, studies using sequencing technology have been utilized in studies involving humans. A linear mixed model analysis using transcriptomic data from TwinUK demonstrated that expression of ECM‐related genes increases with age (Glass et al., 2013). A regression model analysis using GTEx transcriptomic data showed that immune system‐ and mitochondria‐related genes were positively and negatively correlated with age, respectively (Zeng et al., 2020). The ECM, immune system, and mitochondria terms derived from the analysis using TwinUK and GTEx data correspond to the blue, dark red, and black modules derived from WGCNA performed in the present study, respectively. However, previous studies have focused on examining the correlation between individual genes and age without exploring the interrelationships among genes. In the present study, sophisticated network analysis was employed to identify adipose tissue‐specific aging‐related markers. The markers identified through WGCNA are likely to hold functional importance due to their high correlations with one another. Therefore, the hub genes identified in this study are expected to play a more active role in adipose tissue aging.

Compared with the SAT of males or postmenopausal females, the SAT of premenopausal females exhibit metabolically healthier phenotypes; they have a greater abundance of small adipocytes, increased energy expenditure, and higher fat content in the subcutaneous region compared with that in the visceral region (Fitzgerald et al., 2018), indicating sex‐based differences in adipose tissue characteristics. Although we performed consensus WGCNA using separate data for females or males to identify the transcriptomic differences based on sex, a sex‐specific module was not discovered (Figure S4). Therefore, in the present study, we initially analyzed the data without considering the sex of the donors, and subsequently attempted to identify the gene(s) that exhibited differential expression between females and males. Through these analyses, *GYG2* was identified as an aging‐related marker whose expression decreases with age and that is highly expressed in females (Figure 2c). Compared to older females and males of all ages, young females exhibited greater mass and higher activity of beige adipocytes, and accumulated fewer immune cells in SAT (Fitzgerald et al., 2018). These characteristics of young women are very similar to those of the GYG2_H subjects. SAT with high GYG2 expression showed increased mitochondrial activity and lipid metabolism, whereas genes related to inflammation were downregulated. This suggests a close association between GYG2 and adipose tissue characteristics in young female.

In conclusion, the present study revealed that GYG2 is a novel aging‐related marker for SAT. By utilizing diverse bioinformatics tools, we obtained insights into the role of GYG2 in mitochondrial function and brown/beige adipocyte activity. GYG2 not only has the potential to be used in the evaluation of SAT biological age but also offers a valuable lead toward discovering a novel pathway associated with aging. Assuming that GYG2 is proven to be a causal factor of aging, it has the potential to serve as a therapeutic target for anti‐aging treatment. The findings enhance our understanding of the molecular mechanisms underlying SAT aging and may have considerable implications in the development of interventions to promote healthy aging.

## AUTHOR CONTRIBUTIONS

M.H. and K.‐H.K. designed the research; M.H. performed the experiments. M.H., Y.C., T.‐W.K., T.O., H.‐J.K., and K.‐H.K. analyzed and interpreted the results; M.H. and K.‐H.K. drafted the manuscript.

## CONFLICT OF INTEREST STATEMENT

M.H., Y.C., H.‐J.K., and K.‐H.K. are employees of Amorepacific Corporation. T.‐W.K. is an employee in theMoagen. Inc. T.O. has no conflicts of interest.

## PERMISSION STATEMENT

Not applicable.

## Supporting information


Table S1.

Table S2.

Table S3.

Table S4.

Table S5.

Figures S1–S6.

Data S1.

## Data Availability

The data used for the analyses described in this manuscript were obtained from dbGaP (accession number phs000424.v7.p2 on 11/28/2018).
