# Supplementary material for: Transcriptome‐wide analysis reveals GYG2 as a mitochondria‐related aging biomarker in human subcutaneous adipose tissue
Source: Aging Cell. 2023 Dec 8;23(2):e14049. doi: 10.1111/acel.14049 (PMC10861210; doi:10.1111/acel.14049)
Supplement: Supplementary file 6 — Figures S1–S6. [file ACEL-23-e14049-s001.pptx]

## Slide 1
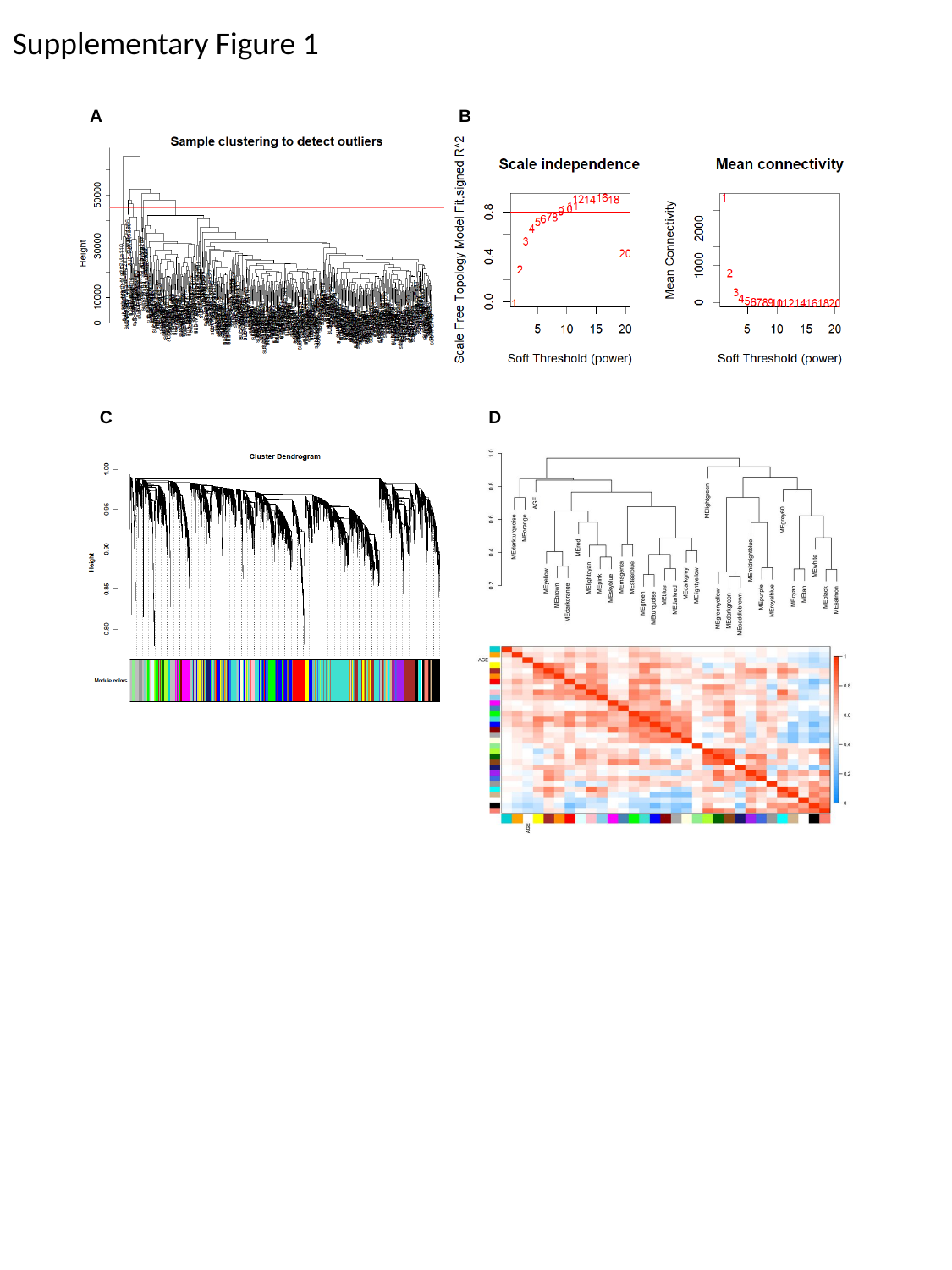

Supplementary Figure 1
A
B
C
D

## Slide 2
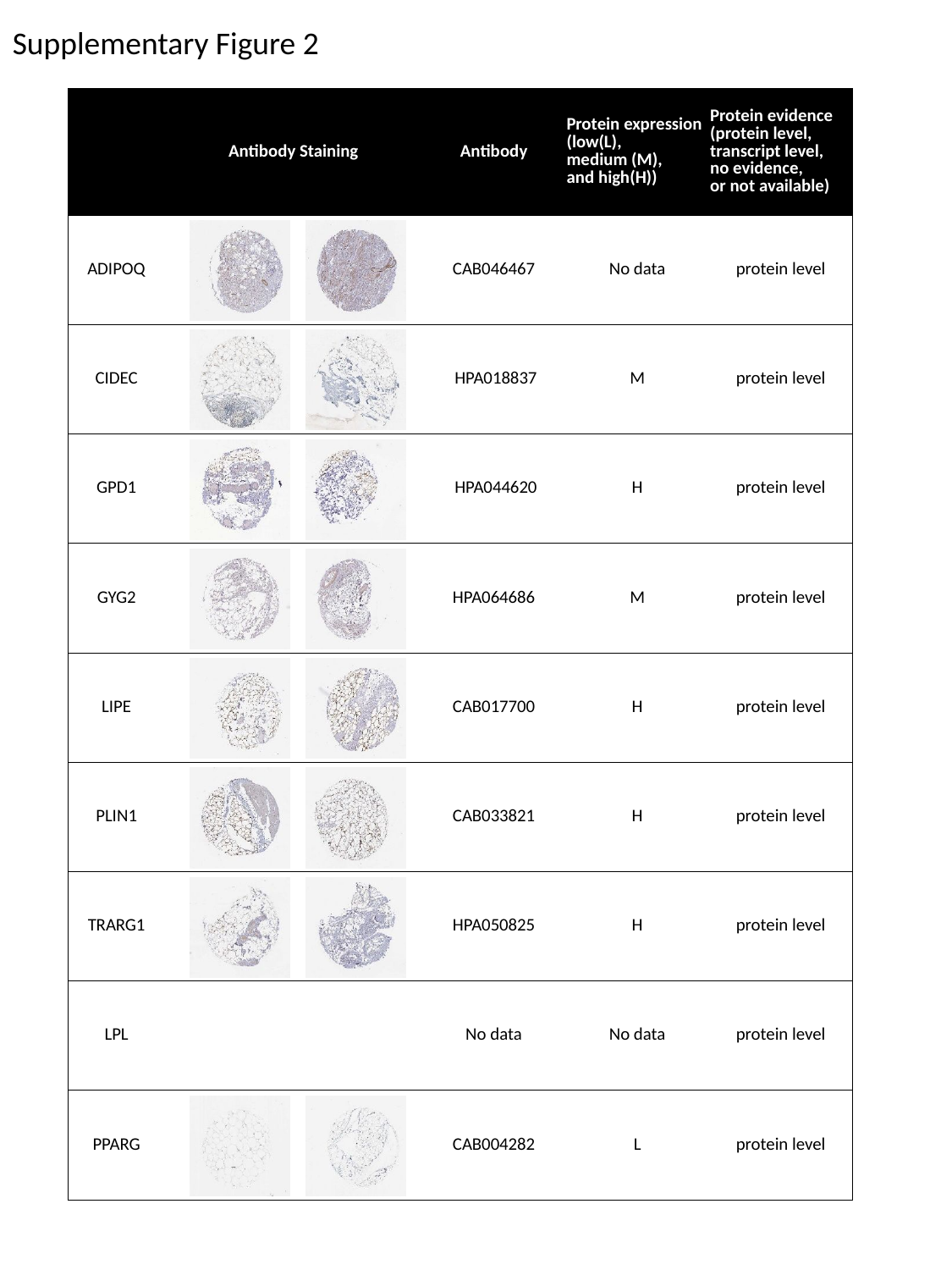

Supplementary Figure 2
| | Antibody Staining | Antibody | Protein expression (low(L), medium (M), and high(H)) | Protein evidence (protein level, transcript level, no evidence, or not available) |
| --- | --- | --- | --- | --- |
| ADIPOQ | | CAB046467 | No data | protein level |
| CIDEC | | HPA018837 | M | protein level |
| GPD1 | | HPA044620 | H | protein level |
| GYG2 | | HPA064686 | M | protein level |
| LIPE | | CAB017700 | H | protein level |
| PLIN1 | | CAB033821 | H | protein level |
| TRARG1 | | HPA050825 | H | protein level |
| LPL | | No data | No data | protein level |
| PPARG | | CAB004282 | L | protein level |

## Slide 3
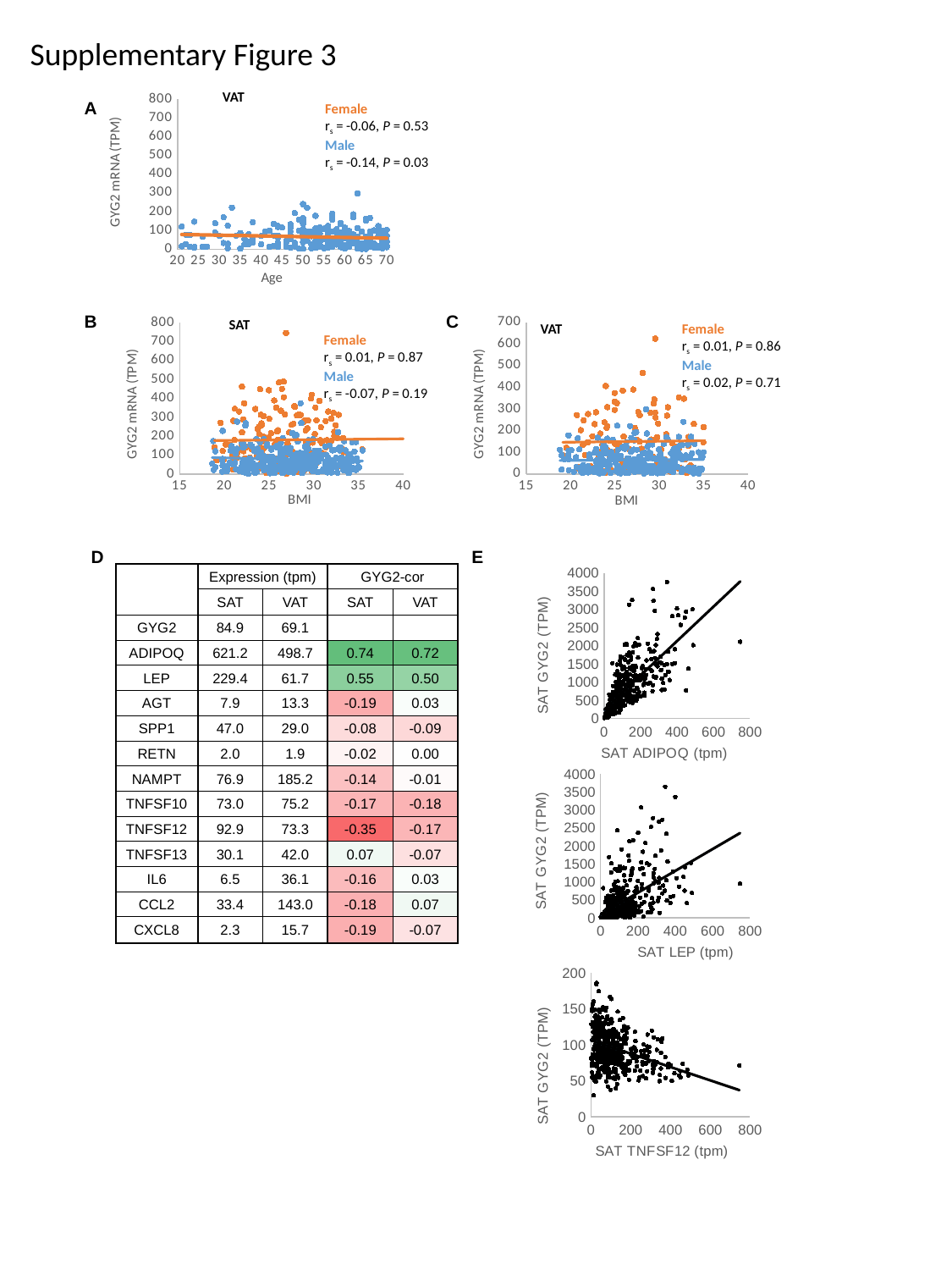

Supplementary Figure 3
VAT
A
### Chart
| Category | vis_female | vis_male |
|---|---|---|Female
rs = -0.06, P = 0.53
Male
rs = -0.14, P = 0.03
B
C
SAT
### Chart
| Category | MALE | FEMALE |
|---|---|---|
### Chart
| Category | MALE | FEMALE |
|---|---|---|VAT
Female
rs = 0.01, P = 0.86
Male
rs = 0.02, P = 0.71
Female
rs = 0.01, P = 0.87
Male
rs = -0.07, P = 0.19
D
E
| | Expression (tpm) | | GYG2-cor | |
| --- | --- | --- | --- | --- |
| | SAT | VAT | SAT | VAT |
| GYG2 | 84.9 | 69.1 | | |
| ADIPOQ | 621.2 | 498.7 | 0.74 | 0.72 |
| LEP | 229.4 | 61.7 | 0.55 | 0.50 |
| AGT | 7.9 | 13.3 | -0.19 | 0.03 |
| SPP1 | 47.0 | 29.0 | -0.08 | -0.09 |
| RETN | 2.0 | 1.9 | -0.02 | 0.00 |
| NAMPT | 76.9 | 185.2 | -0.14 | -0.01 |
| TNFSF10 | 73.0 | 75.2 | -0.17 | -0.18 |
| TNFSF12 | 92.9 | 73.3 | -0.35 | -0.17 |
| TNFSF13 | 30.1 | 42.0 | 0.07 | -0.07 |
| IL6 | 6.5 | 36.1 | -0.16 | 0.03 |
| CCL2 | 33.4 | 143.0 | -0.18 | 0.07 |
| CXCL8 | 2.3 | 15.7 | -0.19 | -0.07 |
### Chart
| Category | |
|---|---|
### Chart
| Category | |
|---|---|
### Chart
| Category | |
|---|---|

## Slide 4
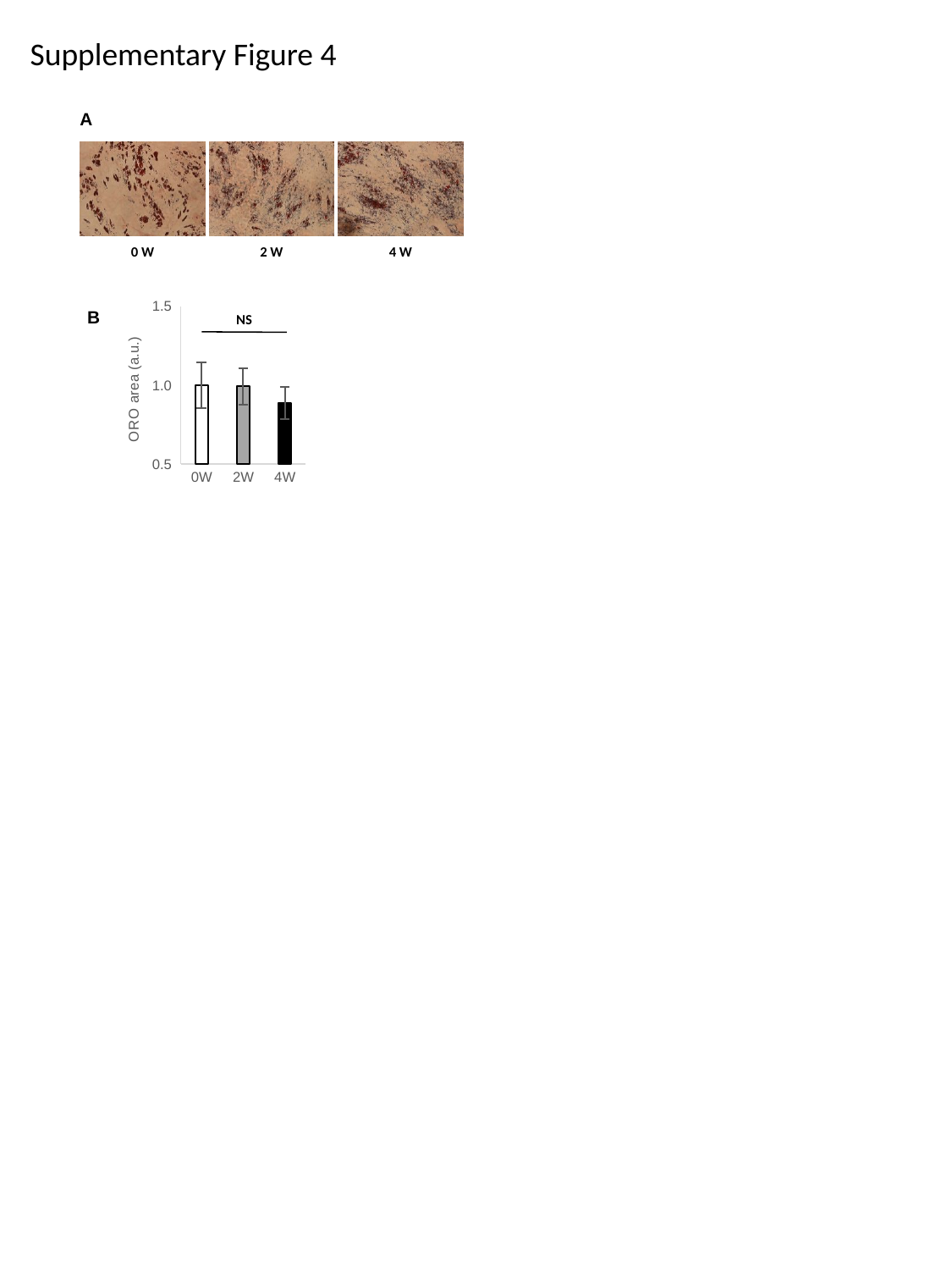

Supplementary Figure 4
A
0 W
2 W
4 W
### Chart
| Category | |
|---|---|
| 0W | 1.0 |
| 2W | 0.9920150090374539 |
| 4W | 0.8853518675286356 |B
NS

## Slide 5
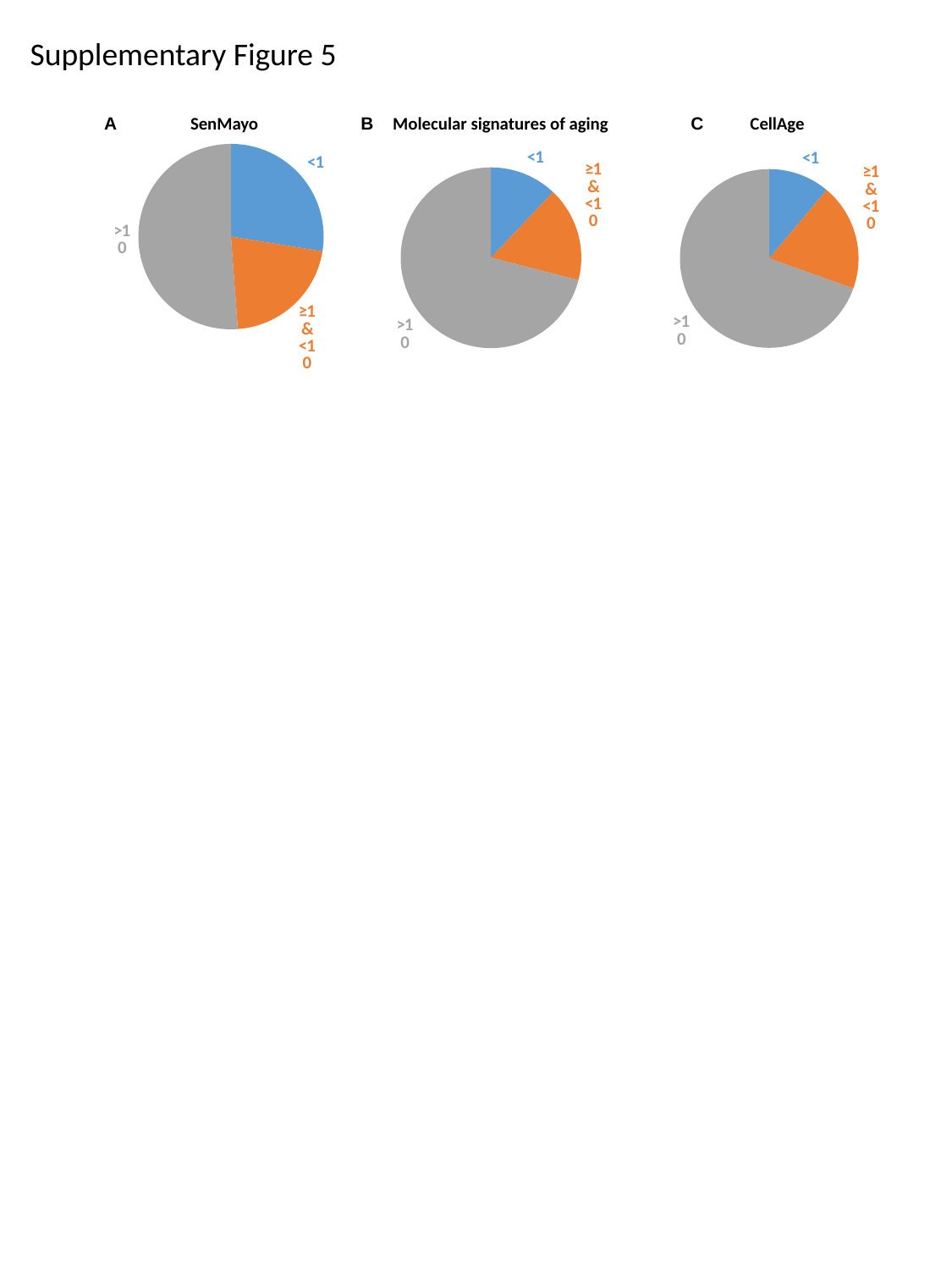

Supplementary Figure 5
A
SenMayo
B
Molecular signatures of aging
C
CellAge
### Chart
| Category | |
|---|---|
| <1 | 35.0 |
| ≥1 & <10 | 27.0 |
| >10 | 65.0 |
### Chart
| Category | |
|---|---|
| <1 | 37.0 |
| ≥1 & <10 | 52.0 |
| >10 | 218.0 |
### Chart
| Category | |
|---|---|
| <1 | 31.0 |
| ≥1 & <10 | 54.0 |
| >10 | 194.0 |

## Slide 6
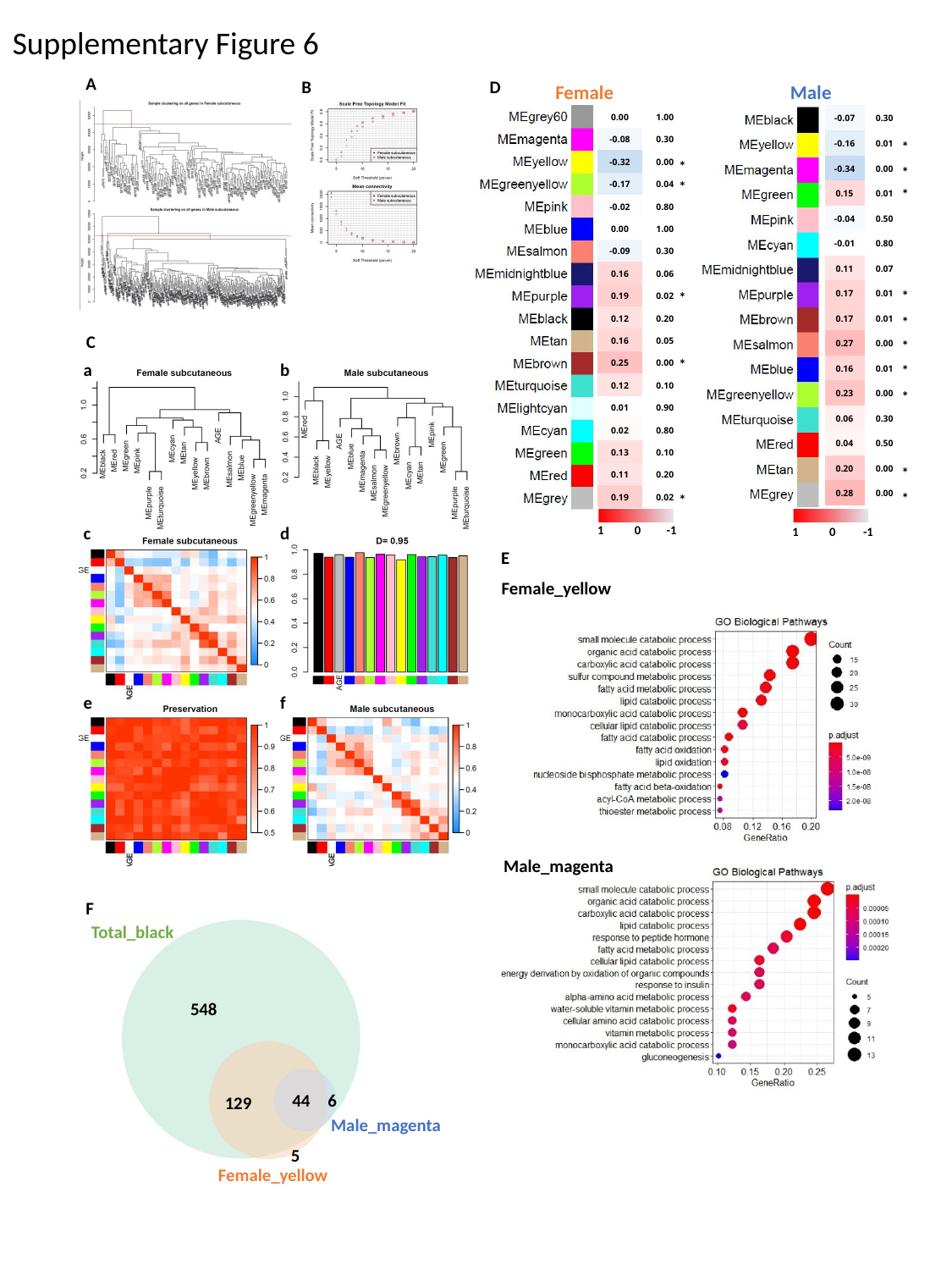

Supplementary Figure 6
A
B
D
Female
Male
| 0.00 | 1.00 |
| --- | --- |
| -0.08 | 0.30 |
| -0.32 | 0.00 |
| -0.17 | 0.04 |
| -0.02 | 0.80 |
| 0.00 | 1.00 |
| -0.09 | 0.30 |
| 0.16 | 0.06 |
| 0.19 | 0.02 |
| 0.12 | 0.20 |
| 0.16 | 0.05 |
| 0.25 | 0.00 |
| 0.12 | 0.10 |
| 0.01 | 0.90 |
| 0.02 | 0.80 |
| 0.13 | 0.10 |
| 0.11 | 0.20 |
| 0.19 | 0.02 |
| -0.07 | 0.30 |
| --- | --- |
| -0.16 | 0.01 |
| -0.34 | 0.00 |
| 0.15 | 0.01 |
| -0.04 | 0.50 |
| -0.01 | 0.80 |
| 0.11 | 0.07 |
| 0.17 | 0.01 |
| 0.17 | 0.01 |
| 0.27 | 0.00 |
| 0.16 | 0.01 |
| 0.23 | 0.00 |
| 0.06 | 0.30 |
| 0.04 | 0.50 |
| 0.20 | 0.00 |
| 0.28 | 0.00 |
*
*
*
*
*
*
*
*
C
*
*
a
b
*
*
*
*
*
c
d
1
0
-1
1
0
-1
E
Female_yellow
e
f
Male_magenta
F
Total_black
548
44
6
129
Male_magenta
5
Female_yellow
