# Supplementary material for: Transcriptome‐wide analysis reveals GYG2 as a mitochondria‐related aging biomarker in human subcutaneous adipose tissue
Source: Aging Cell. 2023 Dec 8;23(2):e14049. doi: 10.1111/acel.14049 (PMC10861210; doi:10.1111/acel.14049)
Supplement: Supplementary file 7 — Data S1. [file ACEL-23-e14049-s006.docx]

Supplementary Table 1. List of utilized TaqMan probes

Supplementary Table 2. GO terms and functional annotations for black, blue and darkred modules

Supplementary Table 3. Differentially expressed genes between GYG2_H and GYG2_L

Supplementary Table 4. Transcription factors in black module genes

Supplementary Table 5. Transcription factors identified using EnrichR
